# Decreasing sperm quality: a global problem?

**DOI:** 10.1186/1471-2458-10-24

**Published:** 2010-01-19

**Authors:** Hiltrud Merzenich, Hajo Zeeb, Maria Blettner

**Affiliations:** 1 Institute of Medical Biostatistics, Epidemiology and Informatics, University Medical Center of the Johannes Gutenberg University, Mainz

## Abstract

**Background:**

Carlsen and coworkers (1992) reviewed 61 heterogeneous observational studies on semen quality published between 1938 and 1990. This review indicates that mean sperm density decreased significantly between 1940 and 1990. An extended meta-analysis with 101 studies confirmed a decline in sperm density for the period from 1934 to 1996 (2000). The key message of the meta-analyses is that sperm counts have decreased globally by about 50% over the past decades. This assessment has been questioned.

**Discussion:**

A major methodological problem of the meta-analysis is the use of data collected in different countries, at different times, on different populations and with different methods of semen analysis. Furthermore, the results of studies concerning semen analysis are frequently biased e.g. by selection criteria of volunteers. In most studies on human semen characteristics the populations under study are insufficiently defined and the study participants are not a representative population sample. The incidence of testicular cancer has increased in Caucasian men worldwide. The investigation of common risk factors for male reproductive disorders requires well designed epidemiological studies and the collection of individual data.

**Summary:**

Former meta-analyses of sperm count data show a global downward trend. This conclusion should be interpreted with caution. The included studies are of great heterogeneity due to geographical and/or ethnical variation, different study designs and different methodological standards. Population-based prospective studies are needed to investigate secular trends in male reproductive disorders.

## Background

Analyses of sperm count data suggest a global downward trend but the results are inconclusive. An increase in male reproductive disorders like cryptorchidism and testicular cancer raise the question of common risk factors. Data on testicular cancer trends are usually based on population-based cancer registries with good validity. The corresponding data from fertility studies need careful consideration in terms of methods and results.

Carlsen et al. [1] reviewed studies on human sperm quality. Publications were excluded if they included men from infertile couples or those referred for oligozoospermia or some genital abnormality. Furthermore, publications were excluded if they included men selected for either high or low sperm count and if counting of sperm cells had been performed with non-manual methods. A total of 61 studies has been included covering results published between 1938 and 1990 without any geographical restriction. The data were analyzed by linear regression in which the weight of each study was dependent on the number of men included in the study. The results of this meta-analysis indicate that mean sperm density decreased from 113 × 10^6^/ml in 1940 to 66 × 10^6^/ml in 1990, and seminal volume decreased from 3.40 ml to 2.75 ml during the same period. These results caused considerable attention. Swan et al. [2] excluded from Carlsens' meta-analysis three non-English-language studies and two studies that included men who conceived only after an infertility work up. In total, 56 studies were reanalysed covering results published between 1938 and 1990. The observed decrease in sperm quality was confirmed. In an extended analysis [3] studies published between 1934 and 1996 were considered which meet the eligibility criteria of Carlsen et al. [1]. In total 19 new studies were found. Furthermore, the authors conducted a further search for the 60-year period 1930-1990 and identified 28 additional eligible studies. Two studies with less than 10 subjects were excluded from the original Carlsen studies. In total, 101 studies were analysed. According to Swan et al. the extended meta-analysis supported a decline in sperm density for the period from 1934 to 1996 [3]. The sperm concentration described in the considered studies varies between 37 × 10^6^/ml and 159 × 10^6^/ml ejaculate fluid. The lowest mean sperm density was found in a study conducted in Brazil among 501 men [4]. The highest mean sperm concentration is derived from an Irish study which of only 10 men [5]. The sample size of the considered studies shows a wide range from 7 [6] to 4435 included men [7].

The central message of the original meta-analysis, that sperm density had decreased globally by about 50% over the past 50 to 60 years, attracted considerable attention and generated much controversy, as well. However, this conclusion should to be interpreted with caution. Reasons for heterogeneity between studies like geographical and ethnical variation, different study designs and different methodological standards have to be considered, as well as selection bias and measurement bias. Important methodological pitfalls in fertility studies will be discussed.

## Discussion

### Geographical and time-related variation

Fisch and co-workers determined whether geographic variations in sperm count might bias the conclusions drawn from studies of semen quality. Briefly, of the 61 studies in the meta-analysis of Carlsen et al. [1] only 20 included more than 100 individuals. A reanalysis of these 20 studies revealed that the majority of studies published before 1970 were from USA, mainly New York. These studies represented locations with the highest sperm counts. In contrast, after 1970, most of the studies were from locations not included earlier. Selection bias due to geographical and ethnic variations could account for the observed decline in sperm quality [8]. However, when the subgroup of studies from the USA (28 studies, including data on 8.329 men) was analyzed separately, a similar trend of decreasing mean sperm values was observed [1]. On the other hand, Lipshultz argued that when all the larger studies containing only New York data are excluded from the meta-analysis, a second linear regression analysis detects no decline in sperm density [9].

The sperm count issue has been extensively investigated since 1992 and the decrease is supported by additional studies. For example, it was reported that sperm counts of 1351 fertile men (semen donors) in Paris decreased by 2.1 percent per year from 89 × 10^6^/ml in 1973 to 60× 10^6^/ml in 1992 (p < 0.001) [10]. Sperm motility also decreased in these individuals. The mean seminal volume was 3.8 ml and did not change during the period from 1973 to 1992. Irvine et al. also examined sperm quality in 571 semen donors in Scotland by birth cohort groups [11]. They reported a significant decrease in median sperm concentration, total number of sperm in the ejaculate and total number of motile sperm in donors born between 1970 and 1974 compared to men before 1959.

On the other hand, several studies confirm that in some locations sperm counts have not decreased over the past 20 to 25 years. More importantly, the results clearly show a remarkable variability in sperm counts at different geographical locations. The mean sperm counts in 302 men in the Toulouse area were unchanged over the period from 1977 to 1992 [12] and their mean sperm count (83 × 10^6 ^ml) was significantly higher than observed in the Paris study [10]. Furthermore, it was reported that the highest sperm counts recorded in Finland were found in men from rural areas accompanied by a low incidence of testicular cancer [13]. These findings suggest that urban lifestyle or environmental factors might be an important etiological factor of testicular malfunction and disease.

Fisch et al. [14] conducted a study comprising 1,283 men who banked sperm before vasectomy in three different sperm banks in the United States from 1970-1994. A slight but significant increase in mean sperm concentration from 77 × 10^6 ^ml to 89 × 10^6 ^ml over the past 25-year period was found. Furthermore, marked differences in semen characteristics between the New York, Minnesota, and California sperm banks were found. Sperm concentration and motility was highest in New York and lowest in California.

It was argued that the data set analyzed by Carlsen et al. [1] was not equally distributed between the decades: 79% of the publications and 88% of the volunteers of all studies were clustered between 1970 and 1990. If joint-analyses were conducted with studies from this period only, an increase in sperm concentrations would become evident. Thus, only 21% of the studies and 12% of the volunteers analyzed before 1970 have caused the regression to have a statistically negative slope [15,16].

Looking at sperm count studies which have been published since 1992, it becomes evident that over the past 20 years, sperm quality has decreased in some but not all locations. The results also show that sperm counts can vary widely between and within countries. These major geographical differences suggest that the results of the former meta-analyses of sperm count may be biased by geographical confounding.

### Selection bias

The WHO recommends that in order to determine normal ranges of semen quality "specimens should be evaluated from men who have recently achieved a pregnancy, preferably within 12 months of the couple ceasing contraception" [17]. In the former meta-analyses [1-3] a precise definition of "proven fertility" is not given. Nevertheless, a significant decline in mean sperm concentration was seen when the 39 papers reporting data on men with "proven fertility" where analyzed separately. In the remaining 22 publications, men were unselected with respect to fertility and were therefore "considered to represent the normal male population".

It is well known that, due to the personal and potentially embarrassing manner of collection, men are reluctant to provide semen samples unless currently concerned about their fertility. In other words, highly selected volunteers constitute a biased sample of the population with regard to their perceived fertility [18]. Furthermore, the socio-demographic profile of sperm donors is commonly shifted to more educated classes [19]. As a result, studies of men providing semen samples involve mainly self-selected volunteers with various non-neutral motivations.

Hence, in most studies on human semen characteristics the populations under study are insufficiently defined and the study participants are not a representative population sample. The former meta-analyses [1-3] included study participants who were examined before vasectomy; some were recruited while their partners were attending antenatal clinics; some were volunteer donors participating in artificial insemination programmes; and some were recruited as part of an occupational study; others were recruited from infertility clinics but were included only if their partners subsequently became pregnant. Data from such study populations cannot be legitimately extrapolated to their apparent source population unless they originate or constitute a representative sample of that reference population.

The "normal male population" included fertile, subfertile as well as infertile men. The lack of external validity of studies using data from self-selected volunteers might further question the hypothesis of a decline in sperm quality with time.

### Confounding bias

Two important factors influencing semen characteristics are age and duration of sexual abstinence (prior to semen donation). Older age contributed significantly to a decline in sperm concentration and sperm motility. The variable for the year of semen donation is composite because it combined each man's age at the time of donation with his year of birth. The relation of each semen characteristic to these independent variables (age at donation and year of birth) has to be tested with multiple regression analysis. Greater sexual abstinence is associated both with age and an increase in the sperm concentration and a decrease in the percentage of motile spermatozoa [10]. In general, sperm concentrations reflect sperm production, age and abstinence time [16]. In the former meta-analyses [1-3], the important information on age was not extracted from the original publications and hence has not been considered in the statistical analysis.

It has been shown that sperm counts in volunteers vary with abstinence time [20]. It has been shown that only 66% of the men investigated had complied with the required abstinence time of three to five days [10]. Thus, if a secular trend in sperm production is to be analyzed, well-controlled abstinence times are a prerequisite.

### Measurement bias

WHO recommends non-manual methods for semen analysis such as haemocytometer or so called counting chambers [21], since manual methods rely on subjective judgements and are not reliable. WHO has laid the foundation for such quality control by introducing a laboratory manual for semen analysis, currently in its fourth edition [22]. WHO recommendations for laboratory techniques are slowly becoming accepted as the international standard, but are not yet universally applied. All data obtained in early studies, as considered in the former meta-analyses [1-3], were not subjected to quality control.

Furthermore, reports on external quality control have shown noticeable variations in the determination of semen characteristics between laboratories related to differences in technique [23]. Consequently, it is very difficult, to compare data from different laboratories if subtle changes in sperm concentration are to be detected [16].

### Statistical considerations

Olsen et al. [24] presented different statistical approaches to the data suggesting that alternative models including the quadratic, spline fit and stair step provided a better fit than the linear regression used by Carlsen et al. and might lead to different interpretations. For example, the use of linear or quadratic models tends to suggest that any change in semen quality is gradual and may be continuing, whereas the stair-step model tends to suggest an isolated event or set of events which is not continuing after say 1970. A sudden apparent fall in semen quality may be due to substantial changes in analytical methodology, subject selection criteria, study selection criteria or widespread introduction of a global environmental factor.

### Goldstandard in measurements of semen quality

The definition of a "normal" sperm concentration has changed from 60 million/ml in 1940 to the present value of 20 million/ml [1,17]. In order to avoid bias, Carlsen et al. restricted their study to men with proven fertility (39 studies) or to "normal" men of unknown fertility (22 studies). It was speculated that much of the apparent change in semen quality could be accounted for by a change in the "accepted" definition of the lower limits of "normal" from around from 60 million/ml in 1940 to values of 20 million/ml accepted today. This change might have led to the exclusion of men with sperm concentration of 20-60 million/ml in the earlier studies, as these studies set out to include "normal men" with sperm counts not below 60 million/ml. This possibility is underlined by a theoretical calculation showing that such a change of the reference data would indeed result in a drop in average sperm concentration. This predicting is in good accordance with the actual data [11,16,25]. However, it has been argued by the authors of the original meta-analysis that at least some of the earlier papers in fact did include men with semen quality in the range below 60 million/ml [26].

Sperm concentration, sperm motility and sperm morphology are related to each other: factors that cause deterioration of one of them usually also have negative impact on the other two [27]. Sperm motility, followed by sperm concentration is the best predictor of fertility [28]. The WHO has given normal limits for semen characteristics. These values are higher than those associated with infertility. They are not based on population samples of fertile men or unselected men, but rather reflect the lower range of men still being fertile [17]. With regard to semen analyses in epidemiological studies the definition of a genuine reference range for human sperm output based on empirical sampling is needed (considering geographical region, ethnicity and age). Furthermore, for clinical or epidemiological studies, it would be preferable to investigate more than one outcome parameter.

### Perspectives

Jorgensen et al. [29] investigated 1082 male partners from pregnant women in four European cities (Copenhagen, Denmark; Paris, France; Edinburgh, Scotland; and Turku, Finland). Semen analysis was standardized, inter-laboratory differences in assessment of sperm concentration were evaluated, and morphology assessment centralized. Lowest sperm concentrations and total counts were detected for Danish men, followed by French and Scottish men. Finnish men had the highest sperm counts. Furthermore, semen quality of a 'standardized' man (30 years old, fertile, ejaculation abstinence of 96 h) was estimated. These data may also serve as a reference point for future studies on time trends in semen quality in Europe. In another study [30] 968 young men from the general population in Denmark, Norway, Estonia and Finland were examined according to the same study protocol. Possible confounders including age of man, year of birth, year of participation in the study, season of year and duration of abstinence were evaluated, and included in the statistical analysis when appropriate. Inter-laboratory differences in assessment of sperm concentrations were controlled by an external quality control programme and morphology assessment was performed by one person. The men examined were recruited from groups attending a compulsory medical examination (military service), and not selected for known fertility or semen quality. Moreover, the majority of participants had no prior knowledge of their fertility potential. This cross-sectional study shows that a geographical gradient exists in the Nordic-Baltic area with regard to semen parameters, somewhat similar to the gradient in incidence of testicular cancer. However, it has to be kept in mind that the participation rate in this study was below 20%.

Carlsen et al. [1] speculate that environmental rather than genetic factors might be responsible for the secular trend in semen quality, which seems to be supported by the observation of increased testicular cancer incidence in many countries [31,32]. However, a possible causal relationship between sperm quality and environmental factors cannot be investigated by description of time-trends and correlation studies. Individual data of both semen quality and data on exposure to environmental factors have to be analysed in analytic case-control or cohort studies. Maternal factors during pregnancy deserve further investigation because they have changed over time and because they might influence male reproductive disorders like cryptorchidism, hypospadias, low sperm counts and testicular cancer [33]. This would correspond to a lifecourse approach regarding the investigation of the semen quality and its determinants. Furthermore, fertility status and semen quality might be predictors of male health and life expectancy [34], which gives an important public health perspective for further studies.

## Summary

The debate on declining semen quality is still ongoing [35,36]. The many studies which examined possible changes in semen quality have produced conflicting conclusions mainly due to the heterogeneity between studies and differences in quality standards. Furthermore, the results of studies are frequently biased e.g. by selection criteria of volunteers or other confounding factors. The observed time trend in semen quality might be an artefact, since the methodological differences between studies might be time dependent as well. Intensive research will be necessary in both clinical and epidemiological domains. More studies are needed with strict methodological standards that investigate semen quality obtained from large samples of healthy men representative for the normal male population.

## Competing interests

The authors declare that they have no competing interests.

## Authors' contributions

All authors made substantial contribution to the conception and design of the paper. HZ revised the paper critically and gave important intellectual input. MB is the principal investigator of the SANCO project and senior author of this manuscript. She contributed to design, analysis and details of the manuscript. All authors read and approved the final manuscript.

## Pre-publication history

The pre-publication history for this paper can be accessed here:

http://www.biomedcentral.com/1471-2458/10/24/prepub
